# HLA-DRB1^*^04 as a Risk Allele to Systemic Lupus Erythematosus and Lupus Nephritis in the Malay Population of Malaysia

**DOI:** 10.3389/fmed.2020.598665

**Published:** 2021-02-10

**Authors:** Malarvili Selvaraja, Voon Kin Chin, Maha Abdullah, Masita Arip, Syafinaz Amin-Nordin

**Affiliations:** ^1^Department of Medical Microbiology, Faculty of Medicine and Health Sciences, Universiti Putra Malaysia, Serdang, Malaysia; ^2^Department of Pathology, Faculty of Medicine and Health Sciences, Universiti Putra Malaysia, Serdang, Malaysia; ^3^Allergy and Immunology Research Centre, Institute for Medical Research, Ministry of Health Malaysia, Kuala Lumpur, Malaysia

**Keywords:** systemic lupus erythematosus, lupus nephritis, HLA-DRB1 gene polymorphism, HLA-DRB1^*^04, Malaysian Malay population, cytokines and free radicals

## Abstract

Systemic lupus erythematosus (SLE) is a chronic autoimmune disease afflicting multiple organs. Lupus nephritis (LN) is a serious complication of SLE and remains a major cause of mortality and morbidity. Curative therapy remains unavailable as etiology from genetic and environmental factors is still unclear. The present study was conducted to elucidate the link between HLA-DRB1 gene polymorphisms with SLE and LN through clinical and laboratory/biological presentations in a population of Malaysian Malay females with SLE. A total of 100 Malay female SLE patients inclusive of 70 SLE patients without LN and 30 patients with LN were included in this study. HLA-DRB1 allele examination in SLE patients was performed using PCR-SSO, and the alleles' frequencies were compared with 951 publicly available datasets representing Malay healthy controls in Malaysia. Cytokines and free radical levels were detected by ELISA and bead-based multiplexed Luminex assays. The association between HLA-DRB1 alleles with clinical and serological manifestations and immune mediators was analyzed using different statistical approaches whenever applicable. Our study showed that HLA-DRB1^*^0405, HLA-DRB1^*^1502, and HLA-DRB1^*^1602 were associated with the increased risk of SLE while HLA-DRB1^*^1201 and HLADRB1^*^1202 alleles were associated with a lower risk of SLE development. Furthermore, HLA-DRB1^*^04 showed significant association to LN and arthritis while HLA-DRB1^*^15 was significantly associated with oral ulcer in Malay SLE patients. Association analysis of HLA-DRB1^*^04 with clinical and biological factors revealed that HLA-DRB1^*^04 was significantly associated with Systemic Lupus Erythematosus Disease Activity Index (SLEDAI) scores, anti-nuclear antibody (ANA), C-reactive protein (CRP) in the blood, and total protein in the urine. SLE carriers with the HLA-DRB1^*^04 allele were significantly correlated to the increased levels of cytokines (IFN-y, GM-CSF, IL-17F, IL-18, IL-21, and VEGF) and were significantly showing negative correlation to IL-5 and free radicals (LPO and catalase enzyme) levels compared to SLE carriers without HLA-DRB1^*^04 allele. The results suggested that disease severity in SLE may be determined by HLA-DRB1 alleles. The risk of HLA-DRB1^*^04 allele with LN was supported by the demonstration of an intense inflammatory response in Malay SLE patients in Malaysia. More studies inclusive of a larger and multiple SLE cohorts in the future are warranted to validate these findings.

## Introduction

Systemic lupus erythematosus (SLE) is a multifactorial, chronic autoimmune disorder that involves multiple organ systems and is predisposed by immunoregulatory, hormonal, environmental, epigenetic, and genetic factors (1). SLE is predominantly characterized by the production of autoantibodies and autoreactive T cells against cytoplasmic, nuclear, and cell-surface antigens (2). Impairment in clearance of apoptotic bodies and nuclear DNA protein immune complexes leads to tissue swelling and induction of inflammation and deposition of immune complexes in multiple organs such as the lung, kidney, brain, skin, and heart, resulting in diversified clinical phenotypes of SLE (2–4). The patterns of autoimmune and clinical manifestations among SLE patients are heterogeneous, which may involve any organ systems in variable combinations, contributing to the variation in disease severity and remain a challenge for accurate diagnosis of SLE (5). Some of the common clinical features present in SLE patients consist of arthritis, mucocutaneous lesions, fever, renal involvement, serositis, hematological disorders, neuropsychiatric dysfunction, and cardiovascular diseases (6). For instance, renal involvement or lupus nephritis (LN) is the most common and severe organ complication of SLE, affecting approximately 30–60% of adults and up to 70% of children with SLE (7). This condition is often associated with high morbidity and poor survival, especially in patients who develop end-stage renal disease (ESRD) (8).

SLE is a global autoimmune disorder with a striking predisposition toward women of reproductive age compared with men. It has been reported that a peak female-to-male ratio of 12:1 is observed during childbearing years. Also, SLE can occur in the elderly and children with a narrower gender distribution (9–11). Presumably, the global incidence and prevalence rates of SLE tend to differ across different geographical regions. Studies have shown that the incidence rates of SLE worldwide range from 0.3 to 31.5 cases per 100,000 individuals while the prevalence of SLE around the world is 3.2–517.5 cases per 100,000 individuals (12). Further, existing literature also documented that racial/ethnicity variations have a huge impact on the incidence and prevalence of SLE. For example, SLE is more common in non-Caucasian populations (African, Asian, Hispanic, and Aboriginal) than in Caucasians (11, 12). Moreover, SLE in these populations exhibits higher disease activity and severity, with heightened risk of relapses and organ damage (13).

Severe disease manifestation such as LN in SLE patients remains a major cause of mortality and morbidity. Much research still needs to be carried out to understand the disease pathogenesis of LN in SLE and to provide alternative treatment. Numerous genomic studies have also highlighted the polymorphisms of various genes at different loci, in particular the major histocompatibility complex (MHC) which encodes the human leukocyte antigen (HLA), cytokines, complement proteins, and immunoglobulin-associated receptor genes and other gene variants that are predisposed to SLE (14–17). Several studies highlighted that renal involvement and severity of disease in SLE were genetically associated. HLA Class II molecules such as HLA-DRB1 and HLADQ genes were predisposed to SLE and LN where the polymorphisms in these genes were widely studied (18–20). HLA-DRB1 and HLA-DQβ1 are considered the most polymorphic with more than 1,000 alleles being discovered at present. The HLA-DR and HLA-DQ genes remain firmly in a state of linkage disequilibrium, and haplotype combinations may modulate the susceptibility to SLE and LN (21).

Ethnicity has been considered as a genetic marker in SLE. Polymorphisms in the HLA-DRB1 gene that affects susceptibility to SLE and class of LN have resulted in different conclusions, depending on the ethnic groups (22–25). Malaysia is a multiracial country consisting of three major ethnic groups, represented by Malay, Chinese, and Indian populations (26). Previous studies reported that the prevalence of SLE is higher in the Chinese population, followed by the Malay and Indian populations (27, 28). Wang and his collaborators (1997) reported that the overall 5-year and 10-year survival rates for SLE patients in Malaysia were 82 and 70%, respectively (28), whereas the mortality rate of SLE patients was 20.2% (29). Renal involvement is the highest among all clinical features in Malaysian SLE patients (28). At present, there are limited studies on HLA-DRB1 in the Malaysian SLE population (30–32). The influence of genetic polymorphisms on clinical manifestation, disease severity, and lab parameters is still unclear. Contributions from immune mediators such as cytokines and free radicals require further investigation. Therefore, the present study aimed to characterize the HLA-DRB1 gene polymorphism and disease susceptibility in Malay SLE population in Malaysia. This study also investigated the association between HLA-DRB1 gene polymorphisms and LN in SLE through clinical, serological, laboratory, and immune mediator association analysis.

## Materials and Methods

### Subjects and Inclusion Criteria

A total of 100 Malay female ages between 18 to 50 years old were recruited from January 2016 to October 2017 for the purpose of this study. Patients with SLE were recruited based on the 1997 American College of Rheumatology (ACR) criteria where SLE is diagnosed based on having at least four of the 11 ACR criteria (33). SLE disease activity in SLE patients was assessed using the Systemic Lupus Erythematosus Disease Activity Index (SLEDAI) (34). All the patients were recruited at the Nephrology Clinic Hospital Serdang, Malaysia. LN was confirmed by a nephrologist by examining the clinical pictures of renal disease/flare, and the classification follows the International Society of Nephrology/Renal Pathology Society (ISN/RPS) 2003 Classification of Lupus Nephritis (35). The clinical manifestations for active nephritis consist of detecting the presence of red blood cells and casts in urine, proteinuria (>2 g), increased levels of creatinine, low complement component C3, and higher levels of anti-dsDNA antibody and erythrocyte sedimentation rate (ESR) (36). All participants gave informed consent, and the study was approved by the Medical Research and Ethics Committee of the Faculty of Medicine and Health Sciences, Universiti Putra Malaysia (UPM), and Medical Research and Ethics Committee (MREC), Ministry of Health Malaysia (NMRR-14-1756-23234).

### Clinical and Laboratory Investigations of Patients With SLE

Demographic data, SLE disease activity, and clinical and laboratory information of patients with SLE were obtained from the Hospital Serdang Information System. Clinical information includes the medical history of patients with SLE, duration of SLE disease, and assessment of clinical features and different organ manifestations. Laboratory information consists of routine blood analysis, urinalysis, and immunological investigations including anti-nuclear antibody (ANA) and anti-dsDNA detection. Other laboratory information such as complement C3, C4, and C-reactive protein (CRP) was also extracted. All the laboratory investigations were conducted by the Medical Lab Technologist of Hospital Serdang, Malaysia.

### Genomic DNA Isolation and HLA-DR Genotyping

Whole-blood samples were collected from 100 Malay female SLE patients in ethylenediaminetetraacetic acid (EDTA) vacutainer tubes. DNA was extracted from whole blood using a QIAamp DNA Blood Mini extraction kit (Qiagen, Germany) in accordance to the manufacturer's instructions. Qualitative and quantitative checks on extracted DNA were performed using a Nanodrop 1000 spectrophotometer (Thermo Scientific, USA). Genotyping of HLA-DR in SLE patients was performed by polymerase chain reaction using sequence-specific oligonucleotides, PCR-SSO (LIFECODES DR-Typing Kit, Gen-Probe, USA) according to the manufacturer's instructions. HLA-DR allele identification was conducted using the Luminex xMAP Technology (R &D Systems, USA) with Lumines 100 IS Software and Quick Type for Life Match 2.6.1 software for Gen-Probe analysis. A total of 951 data consisting of HLA-DRB1 typing representing Malay healthy population were obtained from the Malaysian Stem Cell Registry (MSCR), Institute Medical Research (IMR), Kuala Lumpur, Malaysia (37). The data obtained serve as a control for association analysis between HLA-DR genotyping in SLE Malay female patients with clinical, laboratory, and cytokine indices.

### Association of Cytokines and Free Radicals in HLA-DRB1^*^04 Allele Carrier and Non-carrier

A total of 28 Malay SLE patients (14 SLE patients without LN and 14 SLE patients with LN) and 28 age- and sex-matched healthy controls were used in the association analysis. Cytokines comprised IL-5, IL-17F, IL-18, IL-21, GM-CSF, IFN-γ, and vascular endothelial growth factor (VEGF), and free radicals such as lipid peroxidation (LPO) and catalase (CAT) were used in the association study with HLA-DRB1^*^04 allele SLE carrier and non-carrier SLE patients. Cytokines such as IL-5, IL-17F, GM-CSF, IFN-γ, and VEGF obtained from Human High Sensitivity Cytokine Premixed Performance Kit A and Kit B (R &D Systems, USA) were measured using bead-based multiplexed Luminex assays (R &D Systems, USA) according to the manufacturer's instructions. Cytokine concentrations were detected by Luminex xMAP Technology (R &D Systems, USA), and the results were expressed in pg/mL ± SEM. Concentrations of IL-18 and IL-21 were determined using ELISA kits (EIAab, China) in accordance with the manufacturer's instructions. The absorbance of each supernatants was read at 450 nm using a 96-well microplate reader (Eppendorf, Hamburg, Germany). The detection limits for both cytokines were 15.6–1,000 pg/mL. LPO and CAT activities were assessed through colorimetric measurement. The levels of LPO (Item No. 705002) and CAT (Item No. 707002) purchased from Cayman Chemicals (Ann Arbor, MI, USA) were measured following protocols provided by the manufacturer. The absorbance values of each color compounds in LPO and CAT assays were read at 480 and 540 nm, respectively, using a 96-well microplate reader (Eppendorf, Hamburg, Germany). The assay range for LPO was between 0.25 and 5 nmol while the lowest detection limit for CAT assay was 2 U/mL.

### Statistical Analysis

Data analysis was performed using the SPSS statistical package version 23.0 and GraphPad Prism version 6.0 whenever applicable. The frequency of HLA-DRB1 alleles between SLE patients and healthy controls was compared using the chi-square Fisher test with two-by-two contingency tables used. The chi-square Fisher test was used for the association analysis between HLA-DRB1 alleles with clinical manifestations, disease activity (SLEDAI), and serological manifestations with Phi and Cramer's *V* analysis performed to assess the strength of the association. An independent *t*-test and linear regression were conducted to associate HLA-DRB1 alleles with laboratory findings. An independent *t*-test and correlation analysis were employed to associate HLA-DRB1 alleles with cytokines and free radicals. A *p*-value < 0.05 is considered statistically significant.

## Results

### Demographic, Disease Severity, and Clinical and Laboratory Information of Patients With SLE (With and Without LN Involvement)

A total of 100 Malaysian Malay female patients diagnosed with SLE between January 2016 and October 2017 were included in this cohort study. The demographic and clinical and laboratory findings which were significant and relevant to this SLE study cohort are depicted in Table 1 [part of the data of this SLE study cohort was published (38)]. Among the 100 SLE patients, 70 patients (mean age 31.03 ± 0.95 years) were diagnosed without LN while 30 SLE patients (mean age 29.60 ± 1.23 years) were diagnosed with LN, as confirmed by a nephrologist in Hospital Serdang, Malaysia, by renal biopsy. Additionally, 30 SLE patients were further classified into different classes of LN as shown in Table 1. Majority of the SLE patients without LN were presented with mild activity (mean SLEDAI score 1.97 ± 0.15) in contrast with moderate activity (mean SLEDAI score 9.65 ± 0.57) in SLE patients with LN. SLE patients with LN were quite responsive to anti-dsDNA and ANA detection, with 100 and 93% positivity recorded, respectively, whereas only 80 and 69% positivity for anti-dsDNA and ANA, respectively, were observed in SLE patients without LN. Further analysis from immunological, blood, and urine investigations revealed that SLE patients with LN had significantly lower levels of complement proteins, C3 and C4, and higher levels of creatinine and total protein in urine when compared to SLE patients without LN.

**Table 1:** Demographic, clinical, and laboratory information of patients diagnosed with SLE (with and without LN).

**Category**	**SLE (without LN)**	**SLE-LN (SLE with LN)**	***p*-value**
No of subjects (*N*)	70	30 *Classification of SLE-LN* Class I LN = 2 Class III LN = 6 Class IV LN = 9 Class V LN = 3 Class III + V LN = 8 Class IV + V LN = 2	
Mean age of SLE onset (years ± SEM)	31.03 ± 0.95 (*N* = 70)	29.60 ± 1.23 (*N* = 30)	**ns**
Disease duration (years ± SEM)	7.09 ± 0.62	7.73 ± 0.97	**ns**
SLEDAI activity score			
Mild (0–3)	1.97 ± 0.15 (*n* = 32)	N/A	
Moderate (3–12)	6.61 ± 0.35 (*n* = 28)	9.65 ± 0.57 (*n* = 17)	
Severe (>12)	13 (*n* = 2)	14.23 ± 0.32 (*n* = 13)	
Immunological profiling			
Antinuclear antibodies (ANA) (positive)	69% (*n* = 48)	93% (*n* = 28)	
Anti-dsDNA (positive)	80% (*n* = 56)	100% (*n* = 30)	
Complement (C3) (g/L) (mean ± SEM)	0.462 ± 0.056	0.036 ± 0.028	^*^
Complement C4 (g/L) (mean ± SEM)	0.153 ± 0.017	0.065 ± 0.010	^**^
C-reactive protein (CRP) (mg/L) (mean ± SEM)	8.924 ± 0.401	10.160 ± 0.724	ns
Blood profiling			
Creatinine (μmol/L ± SEM)	273.70 ± 25.990	1,535 ± 262.90	^***^
Urinalysis			
Total protein (Urine) (mg/dl ± SEM)	126.50 ± 7.051	322.60 ± 28.80	^***^

* *indicates p <0.05*,

** *indicates p <0.01*,

*** *indicates p <0.001, ns, not significant*.

### Clinical Manifestations in SLE Patients With and Without LN

**Figure 1:**
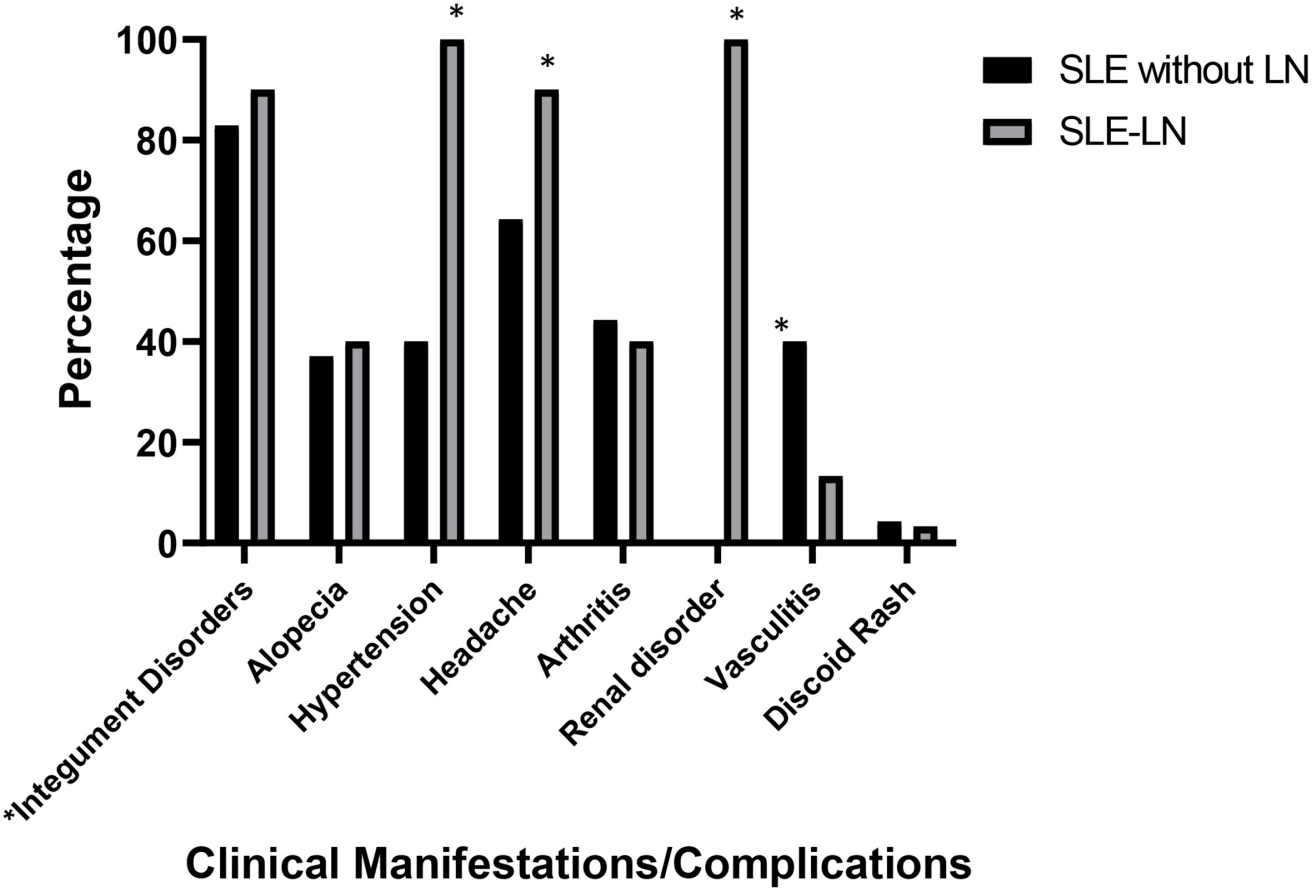
Major clinical manifestations/complications observed in SLE patients with lupus nephritis (SLE-LN) and SLE patients without lupus nephritis (SLE without LN). ^*^Integument disorders comprise clinical features such as oral/nasal ulcers, malar, and photosensitivity. ^*^ denotes *p*< 0.05, significant differences in the clinical manifestations between SLE patients with and without LN.

The main clinical manifestations presented in SLE patients with and without LN are shown in Figure 1 [part of the data of this SLE study cohort was published (38)]. In general, Malay SLE patients without LN were likely presented with milder clinical complications, while Malay SLE patients with LN were more vulnerable to severe clinical manifestations. In SLE patients without LN, 58 patients developed integument disorders (oral/nasal ulcers, malar, and photosensitivity) (82.9%), 45 patients experienced headache (64.3%), 31 patients had arthritis (44.3%), 28 patients developed vasculitis (40%), 28 patients had hypertension (40%), 26 patients had alopecia (37.1%), and three patients had discoid rash (4.3%). In SLE patients with LN, all patients presented with renal disorder and hypertension (100%), whereas 27 patients developed integument disorders (oral/nasal ulcers, malar, and photosensitivity) (90%), 27 patients experienced headache (90%), 12 patients had arthritis (40%), 12 patients had alopecia (40%), four patients developed vasculitis (13.3%), and only one patient had discoid rash (3.3%). Statistical analysis showed that SLE patients with LN had significant clinical presentations which include renal involvement, headache, and hypertension while SLE patients without LN showed significant clinical feature such as vasculitis disorders.

### HLA-DRB1 Genotyping and Allele Frequency in Malay SLE Patients

Thirteen major HLA-DRB1 alleles were identified in Malay SLE patients. The identified HLA-DRB1 alleles and their distribution are shown in Table 2. HLA-DRB1^*^15, HLA-DRB1^*^04, and HLA-DRβ1^*^16 were the most frequent and common alleles among all the HLA-DRB1 alleles identified in SLE patients with and without LN involvement, suggesting a significant role for these alleles in SLE disease. In contrast, the most frequent and common alleles identified in Malay healthy population were HLA-DRB1^*^15, HLA-DRB1^*^12, and HLA-DRB1^*^07 (data not shown). Our analysis also demonstrated the association of SLE disease with 11 different HLA-DRB1 alleles (Table 3). HLA-DRB1 alleles such as HLA-DRB1^*^0405 (OR: 3.493, 95% CI: 2.103–5.801, *p*_c_ = 0.0003), HLA-DRB1^*^1502 (OR = 1.586, 95% CI: 1.132–2.221, *p*_c_ = 0.014), and HLA-DRB1^*^1602 (OR = 2.061, 95% CI: 1.1579–3.668, *p* < 0.05, *p*_c_ = 0.024) were likely associated with the increased risk of developing SLE (with and without LN), while HLA-DRB1^*^1201 (OR: 0.069, 95% CI: 0.017–0.281, *p* = 0.0002) and HLA-DRB1^*^1202 (OR: 0.498, 95% CI: 0.299–0.831, *p* = 0.007) alleles were probably associated with a lower risk of developing SLE (with and without LN).

**Table 2:** HLA-DRB1 allele carrier frequency in Malay female SLE patients and Malay healthy controls.

**HLA-DRB1 allele**	**SLE (2*n* = 200)**	**Controls (2*n* = 1,902)**	**OR CI (95%)**	***p* Fisher**	**p_**c**_**
HLA-DRB1^*^01	4 (0.02)	14 (0.007)	2.752 (0.897–8.443)	ns	ns
HLA-DRB1^*^03	14 (0.07)	96 (0.05)	1.416 (0.792–2.531)	ns	ns
HLA-DRB1^*^04	29 (0.145)	122 (0.064)	2.474 (1.603–3.820)	^***^	(0.001)^***^
HLA-DRB1^*^07	15 (0.075)	192 (0.101)	0.722 (0.418–1.248)	ns	ns
HLA-DRB1^*^08	6 (0.03)	47 (0.025)	1.221 (0.515–2.892)	ns	ns
HLA-DRB1^*^09	7 (0.035)	64 (0.034)	1.042 (0.471–2.305)	ns	ns
HLA-DRB1^*^10	1 (0.05)	37 (0.020)	0.253 (0.035–1.856)	ns	ns
HLA-DRB1^*^11	3 (0.015)	59 (0.031)	0.476 (0.148–1.532)	ns	ns
HLA-DRB1^*^12	19 (0.095)	543 (0.285)	0.263 (0.162–0.426)	^***^	(0.001)^***^
HLA-DRB1^*^13	6 (0.03)	65 (0.034)	0.874 (0.374–2.044)	ns	ns
HLA-DRB1^*^14	3 (0.015)	85 (0.045)	0.326 (0.102–1.039)	ns	ns
HLA-DRB1^*^15	72 (0.36)	503 (0.264)	1.565 (1.152–2.125)	^**^	(0.01)^**^
HLA-DRB1^*^16	21 (0.105)	75 (0.04)	2.858 (1.720–4.748)	^***^	(0.001)^***^

SLE, systemic lupus erythematosus; HLA, human leukocyte antigen; OR, odds ratio; CI, confidence interval;

* *indicates p <0.05*,

** *indicates p <0.01*,

*** *indicates p <0.001, ns indicates no significance*.

**Table 3:** HLA-DRB1 allele carrier subtypes frequency in Malay SLE patients and Malay healthy controls.

**HLA-DRB1**	**SLE (2*n* = 200)**	**Control (2*n* = 1,902)**	**OR CI (95%)**	***P* Fisher**	**P_**C**_**
HLA- DR^*^0401	4 (0.02)	4 (0.002)	9.684 (2.403–39.023)	ns	ns
HLA- DR^*^0403	4 (0.02)	32(0.017)	1.192 (0.4174–3.407)	ns	ns
HLA- DR^*^0405	22 (0.11)	65 (0.034)	3.493 (2.103–5.801)	^***^	^***^(0.0003)
HLA- DR^*^1201	2 (0.01)	242 (0.127)	0.069 (0.017–0.281)	^***^	^***^(0.0002)
HLA- DR^*^1202	17 (0.085)	299 (0.157)	0.498 (0.299–0.831)	^**^	^**^(0.007)
HLA- DR^*^1401	1 (0.005)	23 (0.012)	0.411 (0.055–3.056)	ns	ns
HLA- DR^*^1404	2 (0.01)	56 (0.029)	0.333 (0.081–1.375)	ns	ns
HLA- DR^*^1501	19 (0.095)	125 (0.066)	1.492 (0.900–2.476)	ns	ns
HLA- DR^*^1502	52 (0.26)	345 (0.181)	1.586 (1.132–2.221)	^*^	^*^(0.014)
HLA- DR^*^1601	6 (0.03)	2 (0.001)	29.381 (5.8898–146.57)	ns	ns
HLA- DR^*^1602	15 (0.075)	72 (0.038)	2.061 (1.1579–3.668)	^*^	^*^(0.024)

SLE, systemic lupus erythematosus; HLA, human leukocyte antigen; OR, odds ratio; CI, confidence interval;

* *indicates p < 0.05*,

** *indicates p < 0.01*,

*** *indicates p < 0.001, ns indicates no significance*.

### Association Between HLA-DRB1 Alleles With Clinical Manifestations and Disease Activity in SLE

Associations between HLA-DRB1 with clinical manifestations (based on the 11 ACR Criteria) and disease activity in SLE patients are illustrated in Table 4. HLA-DRB1^*^04 was positively associated with arthritis and renal manifestations. Additionally, HLA-DRB1^*^04 showed a significant and robust positive association with SLE disease activity (SLEDAI, *p* = 0.01) and ANA based on Phi and Cramer's *V* analysis. Also, HLA-DRB1^*^04 was significantly and positively associated with CRP level in the blood and total protein level in the urine (*p* < 0.05) (Table 5). Besides HLA-DRB1^*^04, HLA-DRβ1^*^15 was significantly associated with the increased risk of developing oral ulcer (OR: 5.036, 95% CI: 1.029–24.638, *p* < 0.05).

**Table 4:** Association of the HLA-DRB1 allele with clinical manifestations and disease activity (SLEDAI) in SLE patients.

	**OR (95% CI)**	**Chi-square Fisher**	***p*-value**	**Phi and Cramer's *V***
HLA-DRB1^*^04				
Positive ANA	N/A	4.592	0.032^*^	0.214
Arthritis	2.870 (1.088–7.571)	4.736	0.030^*^	0.218
Renal	5.183 (1.896–14.169)	11.366	0.001^*^	0.337
SLEDAI	N/A	4.717	0.010^*^	0.217
HLA-DRB1^*^15				
Oral ulcer	5.036 (1.029–24.638)	4.697	0.030^*^	0.217

SLE, systemic lupus erythematosus; HLA, human leukocyte antigen; ANA, antinuclear autoantibodies; OR, odds ratio; CI, confidence interval;

* *indicates p <0.05, N/A indicates data are not available*.

**Table 5:** Association of HLA-DRB1^^*^^04 allele with laboratory investigations in Malay SLE patients.

	**Independent samples** ***T*****-test**		**Linear regression**	
	**Mean level in Allele +/- Subjects**	***p*-value**	**Standardized Coefficient (*B*)**	***p*-value**
HLA- DRB1^*^04				
CRP (blood)	10.739/8.889	^*^0.031	0.232	^*^0.030
Total Protein (urine)	242.546/169.167	^*^0.022	0.320	^*^0.018

SLE, systemic lupus erythematosus; HLA, human leukocyte antigen; CRP, C-reactive protein;

* *indicates p < 0.05*.

### Correlation Analysis Between Cytokines and Free Radicals in SLE Carriers With HLA-DRB1^*^04 Allele and SLE Carriers Without HLA-DRB1^*^04 Allele and Healthy Controls

**Figure 2:**
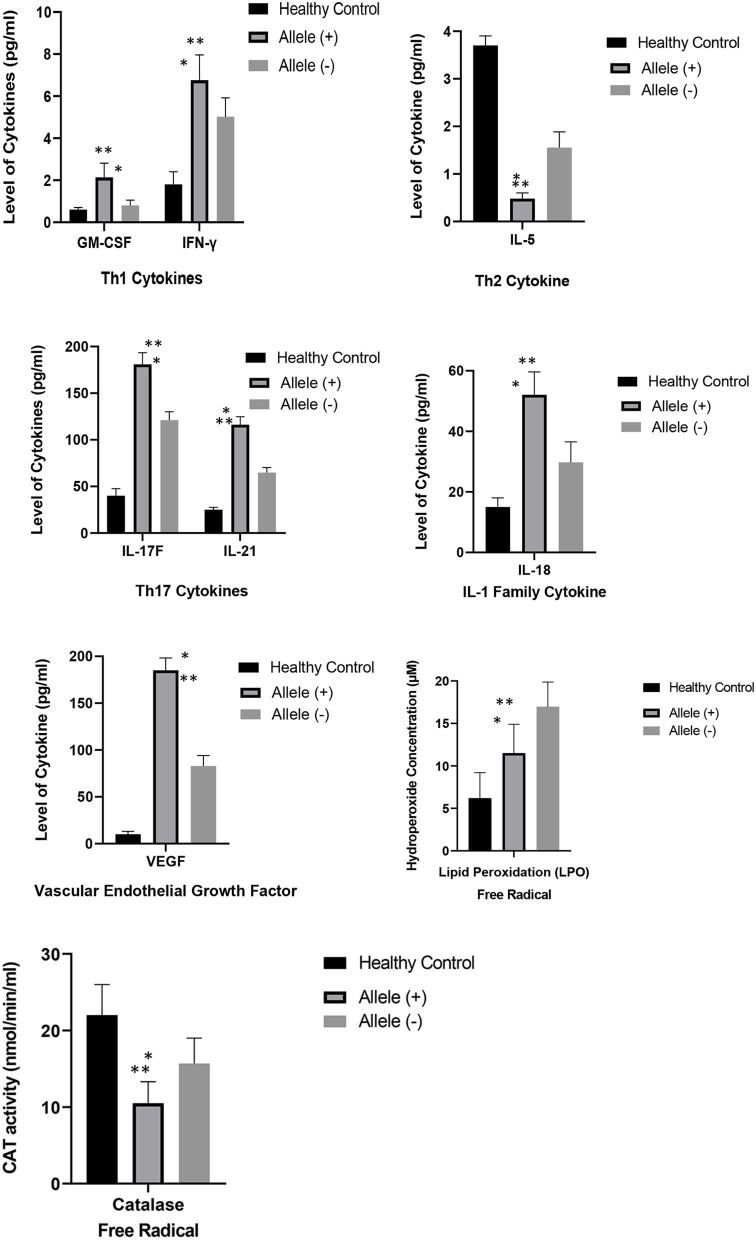
Comparison of the mean levels of cytokines and free radicals between SLE carriers with HLA-DRB1^*^04 allele, SLE carriers without HLA-DRB1^*^04 allele and healthy controls with -age and sex-matched. Results were expressed as mean + SEM of cytokines and free radical levels in eight SLE carriers with HLA-DRB1^*^04 allele, twenty SLE carriers without HLA-DRB1^*^04 allele and twenty-eight healthy controls. Keynotes: Allele (+): SLE carriers with HLA-DRB1^*^04; Allele (–): SLE carriers without HLA-DRB1^*^04. ^*^ denotes *p* < 0.05, significant differences between mean level of cytokines and free radicals between SLE carriers with and without HLA-DRB1^*^04 allele; ^*^^*^ denotes *p* < 0.05, significant differences between mean level of cytokines and free radicals between SLE carriers with HLA-DRB1^*^04 allele and healthy controls with -age and sex-matched.

Figure 2 depicts the correlation between the mean levels of cytokines and free radicals in SLE carriers with HLA-DRB1^*^04 allele and SLE carriers without HLA-DRB1^*^04 allele and age- and sex-matched healthy controls. SLE patients with HLA-DRB1^*^04 allele were significantly correlated to the increased levels of cytokines such as IL-18, IL-21, IL-17F, IFN-γ, and GM-CSF and VEGF compared to SLE patients without HLA-DRB1^*^04 allele and healthy controls. Conversely, a reduction in the levels of IL-5, CAT, and LPO was significantly correlated to SLE patients with HLA-DRB1^*^04 allele in comparison to SLE patients without HLA-DRB1^*^04 allele.

## Discussion

The HLA complex located on the short arm of chromosome 6 (6p21.3), which encodes the MHC proteins in humans, comprises the most polymorphic gene cluster of the whole human genome and has critical roles in regulating the host immune system. Several lines of evidence showed that distinct genes of the HLA complex have significant roles in the modulation of host adaptive immunity (39). Perturbation in the presentation of antigenic peptides by HLA proteins to T cells results in the production of aberrant T-cell-mediated adaptive response, which is why different HLA genes could contribute to the pathogenesis of SLE (40). A plethora of studies have documented the association of gene polymorphisms in HLA-DRB1 allele with SLE and LN. The genetic risk toward the development of SLE and LN is further complicated by the presence of different HLA-DRB1 gene polymorphism profiles across various populations globally (18, 40–42). This warrants a call for an in-depth analysis on the association of HLA-DRB1 gene polymorphism in a specific population to determine the genetic influence in the SLE pathogenesis which could be used to explain the disparity in the clinical phenotypes presented in SLE patients. Therefore, this study was undertaken to investigate the association of HLA-DRB1 gene polymorphism in the development of SLE in Malaysian Malay SLE population. This study also attempts to identify the probable HLA-DRB1 risk alleles associated with LN development in SLE patients through association analysis encompassing clinical, laboratory, and biological factors.

HLA-DRB1 is one of the most critical susceptibility genes in SLE pathogenesis. A recently published meta-analysis that gathered all available case–control studies demonstrated that HLA-DR3 polymorphism is significantly associated with SLE in White populations while HLA-DR15 polymorphism is significantly linked with SLE in Eastern Asian populations. The analysis also reported that the frequencies of polymorphisms in the HLA-DRB1 gene in SLE patients are greatly varied across different ethnicities (43). In the context of the association between SLE and HLA-DRB1 polymorphism in Malay SLE population in Malaysia, our genotyping analysis of the HLA-DRB1 gene showed that HLA-DRB1^*^04, HLA-DRB1^*^12, HLA-DRB1^*^15, and HLA-DRB1^*^16 alleles were significantly associated with SLE development in all Malay female SLE patients (with and without LN involvement). Further analysis revealed that HLA-DRB1^*^0405, HLA-DRB1^*^1502, and HLA-DRB1^*^1602 could be susceptible alleles for SLE development while HLA-DRB1^*^1201 allele could be associated with a lower risk of SLE development. Compared to previous studies conducted in Malaysia, different HLA-DRB1 alleles associated with the risk of SLE development had been identified. For example, HLA-DR2 had been reported to be significantly associated with Malay SLE patients in Malaysia (30, 31). Indeed, HLA-DR2 has been steadily associated with SLE in both Asian and Caucasian populations (44, 45). Another study by Mohd-Yusuf et al. (32) documented that the HLA-DRB1^*^0701 allele could be a risk allele for SLE development, while HLA-DRB1^*^1201, HLA-DRB1^*^1202, HLA-DRB1^*^1203, and HLA-DRB1^*^1301-22 alleles might confer protection in Malay SLE patients, evident by the significant reduction in the allele frequencies of DRB1^*^1301-22 after Bonferroni correction (32). Collectively, studies on HLA-DRB1 gene polymorphism and SLE in different populations in Malaysia are limited, and the validity of the findings could be restricted by small sample sizes. Our findings in this study could enrich the repertoire of HLA-DRB1 alleles that are associated with SLE development in the Malay population in Malaysia. Factors such as sex disparity (46), race (47), and a larger homogenous population (43) should be considered to avoid gender bias, fluctuating effect-size estimates and genetic heterogeneity in determining the association between HLA gene polymorphisms and risk of SLE development.

LN is one of the most severe complications of SLE disease and is a crucial driver of mortality and morbidity in SLE. LN affects 40–70% of SLE patients, with the actual incidence depending on gender, age group, and ethnicity (48). In this single-cohort study, the mean age of SLE onset in SLE patients with LN was 29.60 years. The onset of SLE disease in SLE patients with LN was slightly earlier compared to SLE patients without LN (31.03 years) despite the changes being not significant (Table 1). Similarly, the major clinical presentation in both SLE patient groups (with and without LN) involved integument disorders (oral/nasal ulcers, malar, and photosensitivity). It has been reported that malar rash and photosensitivity are the most common clinical features in other Asian populations as well (49). One study had identified several risk factors predisposed to the development of LN and progression of renal disease to ESRD in SLE patients. These factors include disparity in ethnicity and age, presence of hypertensive condition, and homozygosity for the valine allele of FcγRIIIa (FCGR3A^*^GG) (50). Consistently, our study showed that SLE patients with LN were significantly presented with clinical features such as hypertension, renal disorders, and headache compared to SLE patients without LN. Hence, hypertension in SLE patients could be a probable risk factor in developing LN.

Early detection and prompt diagnosis are imperative since LN is the leading cause of morbidity and mortality in SLE. Delay in diagnosis of LN is a risk factor for the development of ESRD (51, 52). Factors that are associated with impaired renal function such as a rise in total protein concentration in urine and/or increase in serum creatine levels in the blood have been associated with LN and the occurrence of ESRD (53–55). ANA, which is a laboratory hallmark for SLE diagnosis, is also associated with LN (56). Low levels of complements C3 and C4 protein concentrations detected in SLE patients are also highly associated with LN and vasculitis in SLE patients (57). Increased serum levels of CRP in SLE patients are correlated with renal disease activity and increased risk for LN development (58). The decrement in complement levels could probably be due to an increase in immune complex deposition in the kidney, whereas an increase in CRP levels can be associated with increased levels of inflammation (59). Consistent with the aforementioned studies, our study showed that Malay SLE patients with LN had significantly higher levels of serum creatinine in the blood and total protein in urine and lower levels of complements C3 and C4 in comparison to SLE patients without LN. CRP levels in SLE patients with LN were also higher than in SLE patients without LN, although the increment was not significant.

The pathogenesis of LN is due to a loss of immune self-tolerance and subsequent polyclonal antibody activation characterized by full-house nephropathy (concurrent positive staining for IgM, IgA, IgG, C1q, and C3 by immunofluorescence) and positive ANA (60). The pathogenesis could be attributed by genetic variations in humans that encode immune-related functions. These gene variations could disrupt immune tolerance leading to generation of autoantibodies such as anti-dsDNA that might co-opt with genes that are involved in innate immune signaling to produce effector leukocytes and subsequent release of inflammatory cytokines and other autoantibodies that cause renal damage (61, 62). Some genes have been identified in the genesis of LN including HLA-DR, B lymphoid tyrosine kinase (BLK), signal transducer and activator of transcription 4 (STAT4), and toll-like receptor 9 (TLR9) (61, 63). Our association analysis demonstrated that the HLA-DRB1^*^04 allele in Malay SLE patients (with and without LN) was significantly associated with renal disorders and arthritis. However, previous studies showed that the HLA-DR2 allele is positively associated with renal involvement while HLA-DR8 is significantly linked to arthritis in Malay SLE patients (31). Additionally, our findings in this single cohort are also inconsistent with most of the published studies across different geographical regions. Majority of the studies documented that HLA-DRB1^*^15 is a risk allele (18, 64–67) while HLA-DRB1^*^04 is a protective allele for LN (67, 68). One source even claimed that none of the HLA-DRB1 alleles is associated with the risk of LN development among Taiwanese SLE patients (41). Also, a significant association between HLA-DR15-bearing haplotypes with LN in Saudi SLE patients is lacking (69). Although most of the studies relate HLA-DRB1^*^15 with LN, our study had shown that the HLA-DRB1^*^15 allele was significantly associated with oral ulcers in Malay SLE patients. The discrepancies in this study along with diverse association of different HLA-DRB1 alleles with LN could be contributed by genetic heterogeneity and ethnicity, leading to the complexity of different clinical manifestations in SLE patients (70). Consideration on employing genetic polymorphism in HLA-DRB1 gene as a predictor for LN in SLE remains open for debate. A genome-wide association study (GWAS) reported the association of LN with genes outside the MHC region that are more prominent than HLA-DR2 and HLA-DR3. The authors deduce that non-MHC factors may have more profound roles in promoting the development of LN and also that LN loci that influence the kidney response to the immunological aberration caused by SLE might possess higher risk to LN development (71).

The mechanistic aspects between HLA-DRB1 alleles and the risk of LN development remain elusive. Several studies illustrate an association between antibodies anti-Sm/RNP related to DR3 and antibodies against Ro to HLA-DR2 (72–74). Apart from this, Bastian et al. reported a strong association between anti-dsDNA or anti-RNP antibodies and the development of LN in patients already diagnosed with SLE in a European population (64). A comprehensive sequencing analysis of the whole MHC region of a large LN cohort showed that HLA-DRB1 amino acid 11 is one of the five functional risk variants for LN within MHC regions. These independent risk variants also suggest that the risk of development of LN could be due to aberration in peptide presentation by MHC class 1 and 2 molecules to T cells and sex hormone dysregulation (75). Another source presumed that three amino acid positions (11, 13, and 26) located at the HLA-DRB1 epitope-binding groove establish a pathogenic structure in LN patients (76).

Cytokines are small soluble mediators produced by different immune cell subsets, and tissues have shown an undisputable role in regulating the pathogenesis of SLE and severity of SLE disease. The pathogenesis of SLE lies on the imbalance between cytokines released by different T-helper cell subsets which results in immune dysregulation accompanied with elicitation of inflammatory responses and autoimmune abnormalities that cause severe tissue injuries and organ damages as seen in SLE patients (77–79). For example, overproduction of Th1- and Th17-related cytokines could promote T-cell hyperactivity and inflammation in SLE while an excess of Th2-related cytokines typically triggers B-cell hyperactivity and humoral responses (78). On the other side, free radicals and reactive oxygen species (ROS) such as superoxide dismutase (SOD), CAT, nitric oxide (NO), and LPO released by phagocytic cells during inflammation are expected to contribute to tissue injury and disease severity in SLE. The underlying mechanism could be due to deregulation of apoptosis, which leads to a delay in the clearance of apoptotic cells which stimulates the generation of autoantibodies leading to inflammation and severe organ damages (80).

The functional roles of cytokines and free radicals in SLE pathogenesis are well-elucidated. However, limited studies have associated the genetic risk of HLA-DRB1 gene polymorphism with cytokines in the development of SLE and LN. Jacob et al. (68) reported that the HLA-DRB1^*^04 allele confers protection against LN through high levels of TNF-α secretion (68). In this study, Malay SLE carriers with the HLA-DRB1^*^04 allele showed a significant association with the increasing levels of cytokines including IFN-γ, GM-CSF, IL-17F, IL-18, IL-21, and VEGF and a significant negative association with IL-5, LPO, and CAT enzymes. We have performed a literature search and provide a brief summary on the cytokines and free radicals (IFN-γ, GM-CSF, IL-5, IL-17F, IL-18, IL-21, VEGF, LPO and CAT) that are significantly correlated with HLA-DRB1^*^04 allele in Malay SLE patients together with the evidence gathered from a number of previously published studies that depicted the involvement of these cytokines and free radicals in SLE and LN pathogenesis (Table 6). Among all the mediators that are significantly associated with HLA-DRB1^*^04 alelle, IFN-γ, IL-18, VEGF and LPO warrant further investigations as these immune mediators are robustly linked to the pathogenesis of LN in SLE patients (Table 6). Some studies even suggested that IL-18 (95, 97, 98), IFN-γ (81–83), LPO (100) and VEGF (108, 109) could serve as host biomarkers in assessing the renal disease activity and/or discerning between SLE patients with and without lupus nephritis.

**Table 6:** Summary of the association of HLA-DRB1^*^04 allele with cytokines and free radicals (IFN-γ, GM-CSF, IL-5, IL-17F, IL-18, IL-21, VEGF, LPO, and CAT) in Malay SLE patients and the involvement of these cytokines and free radicals in the pathogenesis of SLE and LN based on the findings adapted from different previously published studies.

Association of cytokines and free radicals with HLA-DRB1^*^04 allele in Malaysian Malay SLE Cohort	**Involvement of cytokines and free radicals in SLE patients with and without lupus nephritis**				
	**Th1 subset**	**Th2 subset**	**Th17 subset**	**Other cytokines**	**Free radicals**
Significantly increased in SLE carriers with HLA-DRB1^*^04 allele - IFN-γ and GM-CSF (Th1 subset) - IL-17F and IL-21 (Th17 subset) -IL-18 and VEGF (other cytokines) Significantly reduced in SLE carriers with HLA-DRB1^*^04 allele - IL-5 (Th2 subset - LPO and CAT (Free radicals)	*IFN-**γ*** - Higher level of IFN-γ is associated with SLE patients with severe nephritis and arthritis (81). - Different IFN-γ subtypes could contribute to heterogeneity in clinical manifestations in SLE (81). - Increasing level of IFN-γ is observed in SLE patients which could be potentially serve as a predictor biomarker in SLE diagnosis (82). - Increasing level of IFN-γ is observed in SLE patients that could be applied as a biomarker to assess renal activity (83). - Overexpression of IFN-γ is observed in paediatric SLE patients and IFN-γ is significantly correlated with renal manifestations and SLEDAI score (84). *GM-CSF* - GM-CSF induced by serum juvenile SLE significantly prevent the activation of neutrophil apoptosis and caspase (85). - Neutrophils isolated from SLE patients are more resistant towards apoptosis-inhibiting effects of GM-CSF (86). - GM-CSF secreting peripheral blood mononuclear monocytes (PBMCs) is significantly increased in SLE patients and is correlated with anti-dsDNA (87)	*IL-5* - Increasing serum level of IL-5 is observed in SLE patients (88) -Absence of significant differences between patients with SLE-LN and SLE without LN (88). - Low level of IL-5 is observed in SLE patients and without significant difference between SLE patients and controls (89) - Overexpression of IL-5 is observed in SLE patients with skin lesions (90)	*IL-17* - Increasing serum level of IL-17A in SLE patients with no significant differences is observed between patients with SLE-LN and SLE without LN (88). - Polymorphism of IL-17F rs763780 A/G is associated with susceptibility to SLE in Polish population (91). - IL-17F level is significantly higher in SLE patients which suggests the role of IL-17F in inflammation and angiogenesis (92). - Reduction in the expression of IL17F is observed in SLE T cells in which the reduction is independent of epigenetic pattern activation (93). - Increased in IL-17A/IL-17F ratio may intensify the pro-inflammatory phenotype of SLE (93). *IL-21* - The expression of IL-21 is increased in SLE patients. - IL-21 is positively correlated with SLEDAI score, C3 and erythrocyte sedimentation rate (ES) (94). - Lower serum level of IL-21 is detected in SLE patients compared to healthy controls along with the absence of significant difference in IL-21 level between SLE patients with and without LN.	*IL-18* - IL-18 is significantly increased in active SLE patients and is correlated with SLEDAI score (95). - IL-18 could be a risk predictor of active renal SLE disease (95). - IL-18 showed higher sensitivity and specificity than C3 and anti-dsDNA in predicting active renal and active non-renal SLE cases (95). - Increasing serum level of IL-18 is observed in SLE patients (96). - IL-18 is potentially associated with active renal disease in SLE disease (96). - Higher serum levels of IL-18 is observed in lupus nephritis Class (III, IV and V) than lupus nephritis Class (I and II) (97) - IL-18 is significantly correlated with creatinine and activity index of renal biopsies (97). - IL-18 could inform the extent of renal injury and serve as a potential biomarker to distinguish between the histologic classes of subclinical lupus nephritis (97). - IL-18 level is significantly higher in SLE patients with LN than without LN (98). - IL-18 could be a potential biomarker to assess renal disease in SLE patients (98). - IL-18 is significantly associated with SLEDAI score, proteinuria, renal disease score and disease activity (98). *VEGF* - Increasing serum level of VEGF is observed in SLE patients with and without anti-phospholipid syndrome (99).	*LPO* - Significant differences in the plasma LPO level (measured by the reaction of tiobarbituric acid-reactive substances (TBARS)) is observed between SLE patients with active nephritis, inactive nephritis and non-nephritis (100). - LPO could be one of the potential biomarkers in measuring disease activity of LN (100). - Elevation of LPO, by means of higher malondialdehyde (MDA) level is observed in SLE patients and is associated with disease activity, in particular alopecia and nephritis (101). - Significantly increased in LPO activity, indicated by increased in MDA level is observed in SLE patients (102). - LPO (MDA level) is significantly correlated with SLEDAI score, IFN-γ and IL-12 (102). - Robust positive correlation of MDA and IFN-γ with SLEDAI score suggests the involvement of LPO and pro-inflammatory cytokine in SLE pathogenesis (102). - LPO level, by means of higher MDA level is elevated in SLE patients and is associated with arterial and renal manifestations (103). *CAT* - No significant difference is observed in CAT levels between SLE patients with active nephritis, inactive nephritis and non-nephritis (100). - CAT level is significantly lower in SLE patients compared to healthy controls (104)
			- IL-21 is significantly associated with anemia (105). - Increasing level of IL-21 is observed in PBMCs of SLE patients (106). - IL-21 may synergise with TLR-9 signalling in the production of plasma B cells (106). - Expansion of circulating CD4^+^ T-cells producing IL-21 is observed in SLE patients (107). - IL-21 produced by distinct cellular CD4^+^ T-cell subsets are correlated with T and B cell subsets alterations in SLE (107).	- VEGF is significantly associated with SLEDAI score (99). - Higher level of VEGF expression is significantly associated with SLE patients with LN (108). - Increasing serum level of VEGF is observed in SLE patients with LN (109). - Significant higher level of VEGF is observed in active nephritis than quiescent nephritis (109). - Mild to moderate expression of VEGF is observed in paediatric cases of SLE with lupus nephritis (110).	- CAT level is significantly associated with Arabs, ages ≥40 and SLEDAI score <6 in SLE (104). - Increasing level of CAT is observed in SLE patients (111). - Increasing levels of IgG antibodies (Ab) against CAT is observed in SLE patients (111). - Primary cause of oxidative stress in SLE is due to excessive free radical production rather than impaired CAT activity (111).

Taken together, we surmise that the genetic risk of HLA-DRB1^*^04 for LN could be assessed through different serological manifestations including ANA, CRP, SLEDAI score, and increased proteinuria. We also speculate that intense inflammatory responses regulated by high levels of Th1 (IFN-γ and GM-CSF) and Th17 cytokines (IL-17F and IL-21) along with decreased levels of Th2 cytokine (IL-5) and free radicals (LPO and CAT) contribute to the development of LN in SLE. The expression of these cytokines is more prominent in SLE carriers with the HLA-DRB1^*^04 allele. Nevertheless, further studies are deemed necessary to unravel the actual mechanism inclusive of genetic, environmental, and biological determinants that drives the development of LN in SLE patients. This will eventually pave a path to uncover the genesis of LN and its pathogenesis and facilitate the identification of predictive biomarkers in the evaluation of disease activity and treatment intervention.

In conclusion, there are some limitations in our study. The findings from this study were solely based on 100 Malay female SLE patients with the HLA genotype, and the association analyses with clinical, serological, and laboratory manifestations were compared with the publicly available dataset. A larger SLE cohort considering sex and ethnicity is required to untangle the genetic basis of SLE and LN in a multiracial country like Malaysia. The environmental, sociodemographic, and epigenetic influences should be carefully assessed as well in SLE. Despite these limitations in our study, at least in the Malay SLE cohort in Malaysia, the HLA-DRB1^*^04 allele could be associated with susceptibility to SLE and LN. We agree that more biological studies are needed to validate and to confirm these associations and to explain discrepancies in different populations. Further, the significant correlation of HLA-DRB1^*^04 allele with specific cytokines and free radicals suggests that specific HLA molecules may significantly influence cytokine responses to antigenic stimulation and immune outcomes. These cytokines could serve as a panel of biosignature to assess the disease severity and biomarker in a specific population.

## Data Availability Statement

The data presented in the study are included in the article, further inquiries can be directed to the corresponding author/s.

## Ethics Statement

The studies involving human participants were reviewed and approved by the Medical Research and Ethics Committee of the Faculty of Medicine and Health Sciences, Universiti Putra Malaysia (UPM) and Medical Research and Ethics Committee (MREC), Ministry of Health Malaysia (NMRR-14-1756-23234). The patients/participants provided their written informed consent to participate in this study.

## Author Contributions

MS carried out the whole SLE study and the analysis of the data. VKC analyzed data, wrote and reviewed the first draft and final version of the manuscript. SA-N and MAb were involved in the conception, funding acquisition, designation of the study, supervision, and analysis of the data and made substantial contributions to the analyses and review of the manuscript. MAr was involved in the supervision and analysis of the data. All authors read and approved the final version of the manuscript.

## Conflict of Interest

The authors declare that the research was conducted in the absence of any commercial or financial relationships that could be construed as a potential conflict of interest.
